# Effect of planting patterns on yield, nutrient accumulation and distribution in maize and soybean under relay intercropping systems

**DOI:** 10.1038/s41598-019-41364-1

**Published:** 2019-03-20

**Authors:** Muhammad Ali Raza, Muhammad Hayder Bin Khalid, Xia Zhang, Ling Yang Feng, Imran Khan, Muhammad Jawad Hassan, Mukhtar Ahmed, Muhammad Ansar, Yuan Kai Chen, Yuan Fang Fan, Feng Yang, Wenyu Yang

**Affiliations:** 1 College of Agronomy, Sichuan Agricultural University, Chengdu, Sichuan 611130 China; 2 China Key Laboratory of Crop Eco-physiology and Farming System in Southwest, Ministry of Agriculture, Chengdu, 611130 P.R. China; 3 Maize Research Institute, Sichuan Agricultural University, Chengdu, Sichuan 611130 PR China; 4 Department of Grassland Science, Sichuan Agricultural University, Chengdu, 611130 PR China; 5 Department of Agronomy, Pir Mehr Ali Shah Arid Agriculture University, Rawalpindi, Punjab Pakistan

## Abstract

Planting patterns affect nitrogen (N), phosphorus (P), and potassium (K) acquisition and distribution in maize and soybean under intercropping conditions. Here we reveal that strip relay-intercropping increases the N, P, and K uptake and distribution across plant organs (root, straw, and seed) of maize and soybean, accelerates the dry-matter production of intercrop-species, and compensates the slight maize yield loss by considerably increasing the soybean yield. In a two-year experiment, soybean was planted with maize in different planting patterns (SI, 50:50 cm and SII, 40:160 cm) of relay-intercropping, both planting patterns were compared with sole cropping of maize (SM) and soybean (SS). As compared to SI, SII increased the N, P, and K accumulation in each organ of soybean by 20, 32, and 18 (root) %, 71, 61, and 76 (straw) %, and 68, 65, and 62 (seed) %, respectively, whereas decreased the N, P, and K accumulation in each organ of maize by 1, 4, and 8 (root) %, 1, 10, and 3 (straw) %, and 5, 10, and 8 (seed) %, respectively. Overall, in SII, relay-cropped soybean accumulated 91% of total nutrient uptake (TNU) of sole soybean plants, and relay-cropped maize accumulated 94% of TNU of sole maize plants.

## Introduction

Relay-intercropping systems are used worldwide since these can increase the land use efficiency and nutrient use efficiency^1^. It is important and continues to be broadly practiced not only under tropical regions^2^, but in temperate regions also^3^, resulting in higher seed yields^4^. The maize soybean relay-intercropping is the major planting system, especially under rainfed regions^5–7^. As compared to intercropping systems, the advantage of relay-intercropping system is higher because in intercropping systems both crops almost have the similar growth periods and they required high amount of inputs to produce higher intercrop yields, whereas under relay-intercropping system both crop species have different growth periods and have complementary resource use in time. In addition, for maize soybean relay-intercropping system (MS_R_), the land equivalent ratio (LER) often reaches 1.7–1.8 when both crops were planted at their optimal planting density^8^, which increases its popularity among farmers, especially among small farmers. In MS_R_, spring maize is sown as two rows narrow strips in the first or second week of April, while two rows of soybean are sown in the fellow wide rows in the mid of June. Maize is subsequently harvested in the mid of August and soybean is harvested in the first week of November^9^. Previously, researchers have obtained higher values of LER under MS_R_^8,10,11^. The reasons for high LER are as follows: (a) increase in the gap between maize and soybean rows by narrowing the distance between maize rows^12^; (b) use of similar planting density between sole and intercropping system by adjusting the plant distance (smaller distance) in every row under intercropping conditions, which compensates for decreased row number^10^; and (c), optimum light distribution in maize and soybean rows by developing compact varieties of maize which enables sun light to reach maximum at soybean canopy^13^. Presently this system has been used in many provinces of China, such as Ningxia, Henan, Guizhou, Shandong, Sichuan, etc.^14,15^. The maximum intercrop yields in every province were utilized for comparison to estimate LER, e.g., 9000 and 3000 kg ha^−1^ for maize and soybean, respectively in Shandong province. This method reached LER of 1.8 under relay-intercropping system^11^, which is much higher as compared to the averaged value of LER 1.22 ± 0.02^16^ and 1.30^17^. In relay-intercropping system, maize had a yield advantage due to the better growth and border rows effect, and almost produced equal or higher seed yield than sole maize yield^11^, whereas soybean is the later grown crop of this system, as the result of crop competition with maize, the early growth of soybean is slower than sole soybean^18^. Previously, it has been reported that during the co-growth period soybean suffered from heavy maize shading which negatively affected the plant morphology^19^, physiology^13^ and carbon production^20^. In addition, relay-intercropped soybean attained lower photosynthetic rate^21^ and biomass per plant than sole cropped plants^22^. However, soybean plants exhibited fast recovery growth after the harvest of maize crop, and the severe competition for light and nutrients from maize plants during the co-growth period is compensated after the maize harvesting because the ability of soybean recovery growth was found to be stronger in MS_R_^23^. Moreover, in later stages of growth, soybean under relay-intercropping system has the better access to land, nutrients, light, and water resources per plant in comparison with sole cropping system^8^. In past, it has been found that the stronger recovery growth of relay intercropped crop species related to higher nutrient uptake^24^ and radiation use efficiency of soybean plants under relay-intercropping system^14^. In addition, this type of strong competition during the co-growth period and then relaxed competition during the later growth stages recognized as ‘competition recovery production principle^25^.

In previous studies, scientists have mainly focused on the morphological characteristics and seed yield of intercrop species under different planting (plant density and row spacing) arrangements^5,26^. However, no experiment has been carried out to investigate the major nutrient (nitrogen, phosphorus, and potassium) uptake and distribution among different plant parts of maize and soybean under different planting patterns in MS_R_. Some researchers have documented the nitrogen (N) and phosphorus (P) uptake in maize under wheat-maize relay intercropping system under different row configuration^27^, but the effects of different planting patterns on nutrient uptake and distribution in maize and soybean are not clearly understood. Therefore, it is important to investigate the nutrient uptake and distribution pattern in maize and soybean for sustainable agriculture production. In addition, relay-intercropping systems are different from the intercropping systems in co-growth periods of intercrop species, and planting pattern plays a vital role in increasing the intercrop advantage^28^. Up till now, the nutrient uptake and distribution pattern among different plant parts of maize and soybean under MS_R_ remain unclear. Therefore, investigating the effects of different planting patterns on nutrient uptake and distribution in intercrop species is important in making nutrient decisions for intercrop species and to maintain agriculture sustainability in MS_R_. This experiment aims (i) to investigate the effects of different planting patterns on nutrient uptake and distribution in maize and soybean at plant and organ level; (ii) how increase in nutrient uptake (N, P, and K) affects the dry matter production and yields of maize and soybean under different planting patterns; (iii) to suggest an appropriate planting pattern which is better in utilizing the nutrients (N, P, and K) and resources with respect to competition and productivity.

## Results

### Dry matter accumulation and partioning

**Figure 1:**
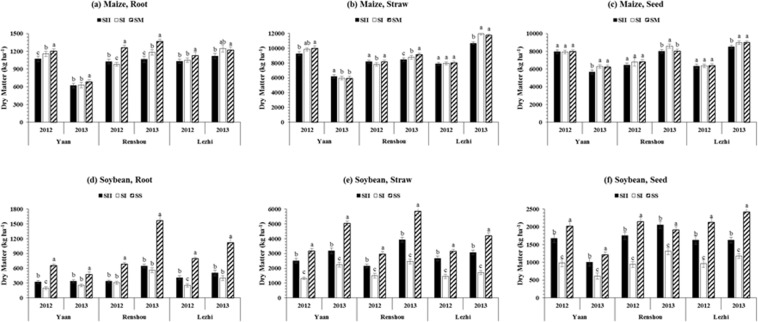
Dry matter distribution in root, straw, and seed of maize and soybean at various locations (Yaan, Renshou, and Lezhi) under different planting patterns. The SI (50 cm: 50 cm) and SII (40 cm: 160 cm) represent the different planting patterns under relay-intercropping system. The SM and SS refer to sole cropping system of maize and soybean, respectively. Means are averaged over three replicates. Bars show ± standard errors, (n = 2). Within a bar, different lowercase and same letters show a significant and non-significant difference (p ≤ 0.05) between treatments.

The mean values for dry matter accumulation (DMA) showed that different planting patterns and locations had significant impact on DMA of maize and soybean (Table 1). In our study, plants in sole maize (SM) and sole soybean (SS) always accumulated higher dry matter than those under SI (single row relay-intercropping system of maize and soybean) and SII (double row relay-intercropping system of maize and soybean) in MS_R_. However, the planting pattern, SI (17244 kg ha^−1^) and SII (4983.1 kg ha^−1^), and location Lezhi (18431 kg ha^−1^) and Renshou (5515.2 kg ha^−1^) produced the highest dry matter of maize and soybean, respectively. Furthermore, the DMA of maize showed the trend planting pattern, SM > SI > SII > and locations, Lezhi > Renshou > Yaan, and in soybean it showed planting pattern, SS > SII > SI > and locations, Renshou > Lezhi > Yaan, suggesting that increasing the distance between maize and soybean rows (planting pattern, SII) under MS_R_ significantly increased the DMA of soybean by reducing the negative effects of maize shade on soybean growth. For instance, for treatment SII, it increased the DMA of soybean by 61% as compared to SI treatment. Moreover, the different planting patterns and locations significantly changed the pattern of dry matter partitioning among different plant parts of maize (Fig. 1a–c) and soybean (Fig. 1d–f). At maturity of maize and soybean, the maximum partitioning of dry matter was measured in straw followed by seed and root under all the treatments. Importantly, between SI and SII treatments in MS_R_, the soybean seed dry matter, maximum (1624.8 kg ha^−1^) was found under SII, while minimum (999.9 kg ha^−1^) was noted in SI. Among the locations, highest soybean seed dry matter (1689.3 kg ha^−1^) was recorded at Renshou, whereas, lowest (1252.4 kg ha^−1^) was measured at Yaan in both years. In addition, the planting pattern, SI (7485.7 kg ha^−1^) and location, Lezhi (7593.2 kg ha^−1^) produced the highest maize seed dry matter, while lowest seed dry matter of maize was found under SII (7150.1 kg ha^−1^) at Yaan (7007.1 kg ha^−1^). On average, treatment SII significantly increased the dry matter allocation to soybean seed by 41, 46, and 40% in 2012 and 38, 36, and 27% in 2013 at Yaan, Renshou, and Lezhi, respectively than those of under SI treatment.

**Table 1:** Effect of different planting patterns on dry matter accumulation (DMA, kg ha^−1^), seed yield (kg ha^−1^), and harvest index (HI, %) of maize and soybean at different locations under relay intercropping system.

Years	Treatments		Maize			Soybean		
			DMA	Seed Yield	HI	DMA	Seed Yield	HI
2012	Planting Patterns	SI	16621b	7020	42	2638c	962c	37
		SII	16416b	6913	42	4513b	1684b	37
		SM	16984a	7076	41	—	—	—
		SS	—	—	—	5900a	2095a	36
	LSD (0.05) Locations		340.00	NS	NS	67.21	132.68	NS
		Yaan	18803a	7953a	42a	4283b	1627	37
		Renshou	15833b	6682b	42a	4291b	1571	37
		Lezhi	15385b	6363c	41b	4476a	1534	36
	LSD (0.05)		590.04	234.13	0.57	64.74	NS	NS
2013	Planting Patterns	SI	17867a	8447a	48	3550c	1022c	29b
		SII	16753b	7957b	48	5452b	1758b	32a
		SM	17760a	8491a	49	—	—	—
		SS	—	—	—	7939a	1928a	25c
	LSD (0.05)		602.35	103.14	NS	72.88	56.24	1.64
	Locations	Yaan	12714c	7228c	56a	4788c	1442b	31a
		Renshou	18190b	7988b	43b	6738a	1652a	26b
		Lezhi	21477a	9680a	45b	5415b	1614a	25c
	LSD (0.05)		667.71	234.47	1.37	89.80	106.48	1.48

The SI (50 cm: 50 cm) and SII (40 cm: 160 cm) represent the different planting patterns under relay-intercropping system. The SM and SS refer to sole cropping system of maize and soybean, respectively. Means are averaged over three replicates. Means do not share the same letters in the column differ significantly at p ≤ 0.05; NS = Non-significant.

### Root, straw, and seed nitrogen (N) concentration of maize and soybean

**Figure 2:**
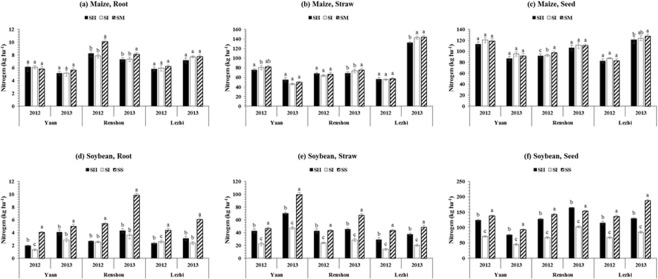
Nitrogen allocation in root, straw, and seed of maize and soybean at various locations (Yaan, Renshou, and Lezhi) under different planting patterns. The SI (50 cm: 50 cm) and SII (40 cm: 160 cm) represent the different planting patterns under relay-intercropping system. The SM and SS refer to sole cropping system of maize and soybean, respectively. Means are averaged over three replicates. Bars show ± standard errors, (n = 2). Within a bar, different lowercase and same letters show a significant and non-significant difference (p ≤ 0.05) between treatments.

Different planting patterns and locations significantly (p ≤ 0.05) affected the total nitrogen accumulation (TNA) in maize and soybean. The mean maximum TNA was 190.9 and 141.7 kg ha^−1^ under SM and SS, 208.8 and 126.4 kg ha^−1^ at Lezhi and Renshou, respectively in both study years (Table 2). Concentration of N among different plant parts of maize and soybean is presented in Fig. 2 for all planting patterns and locations. In different relay-intercropping planting patterns, the root, straw and seed concentration in maize decreased as the distance between maize and soybean rows increased under MS_R_, while the opposite results were noticed for soybean. Root (7.3 and 3.1 kg ha^−1^), straw (79.1 and 44.5 kg ha^−1^) and seed (104.6 and 122.9 kg ha^−1^) N concentration of maize and soybean in SM and SS, respectively was higher than the corresponding values under SI and SII treatments. In all treatments (i.e., different planting pattern and locations), seed and straw of maize (Fig. 2a–c) and soybean (Fig. 2d–f), respectively had significantly higher while root had significantly (p ≤ 0.05) lower nitrogen concentration in both years. Locations had significant and non-significant effects on root and straw, and seed N concentrations of maize. In maize, the mean highest root (8.1 kg ha^−1^) and straw (97.9 kg ha^−1^) N concentration was measured at Renshou and Lezhi, respectively, whereas mean lowest N concentration in root (5.7 kg ha^−1^) and straw (64.8 kg ha^−1^) was observed at Yaan in both years. Furthermore, the average maximum root (4.7 kg ha^−1^) and seed (126.4 kg ha^−1^), and straw (54.7 kg ha^−1^) N concentration in soybean were determined at Renshou and Yaan, respectively as compared to other locations. Overall, soybean plants accumulated 68, 69, and 64% higher N in SII than SI at Yaan, Renshou, and Lezhi, respectively.

**Table 2:** Effect of different planting patterns on total nitrogen accumulation (TNA, kg ha^−1^), total phosphorus accumulation (TPA, kg ha^−1^), and total potassium accumulation (TKA, kg ha^−1^) in maize and soybean at different locations under relay intercropping system.

Years	Treatments		Maize			Soybean		
			TNA	TPA	TKA	TNA	TPA	TKA
2012	Planting Patterns	SI	173.1ab	34.1a	260.9b	90.6c	16.8c	29.9c
		SII	169.1b	29.8b	246.1c	162.8b	29.1b	58.5b
		SM	175.5a	30.9b	271.8a	—	—	—
		SS	—	—	—	187.7a	36.8a	72.2a
	LSD (0.05)		4.65	1.28	4.43	3.32	1.41	2.75
	Locations	Yaan	202.6a	39.3a	297.4a	150.2b	27.1b	54.9
		Renshou	168.7b	30.8b	243.8b	152.9a	28.0b	53.8
		Lezhi	146.4c	24.7c	237.5c	138.1c	28.6a	51.9
	LSD (0.05)		6.52	1.50	3.23	2.45	0.82	NS
2013	Planting Patterns	SI	204.4a	34.9a	169.6b	112.5c	12.5c	59.5c
		SII	196.3b	32.9b	155.4b	178.2b	18.7b	92.2b
		SM	206.3a	34.6a	173.7	—	—	—
		SS	—	—	—	223.3a	26.1a	128.2a
	LSD (0.05)		4.57	0.98	3.20	2.70	0.86	2.02
	Locations	Yaan	146.7c	28.4c	108.2c	147.7c	14.5b	64.5c
		Renshou	189.3b	39.7a	221.2a	173.2a	21.1a	125.1a
		Lezhi	271.2a	34.3b	180.3b	173.1b	21.8a	90.3b
	LSD (0.05)		5.51	2.61	4.95	2.63	1.50	2.41

The SI (50 cm: 50 cm) and SII (40 cm: 160 cm) represent the different planting patterns under relay-intercropping system. The SM and SS refer to sole cropping system of maize and soybean, respectively. Means are averaged over three replicates. Means do not share the same letters in the column differ significantly at p ≤ 0.05; NS = Non-significant.

### Root, straw, and seed phosphorus (P) concentration of maize and soybean

**Figure 3:**
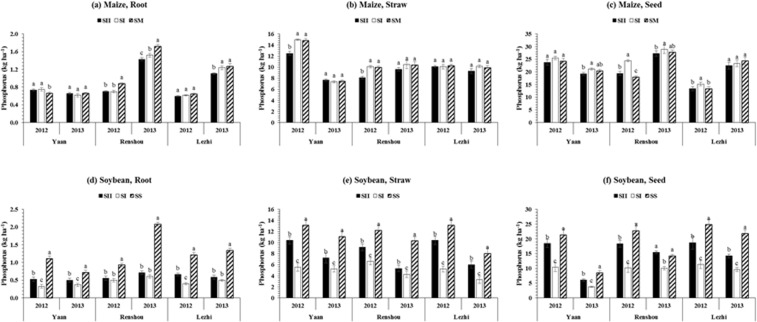
Phosphorus allocation in root, straw, and seed of maize and soybean at various locations (Yaan, Renshou, and Lezhi) under different planting patterns. The SI (50 cm: 50 cm) and SII (40 cm: 160 cm) represent the different planting patterns under relay-intercropping system. The SM and SS refer to sole cropping system of maize and soybean, respectively. Means are averaged over three replicates. Bars show ± standard errors, (n = 2). Within a bar, different lowercase and same letters show a significant and non-significant difference (p ≤ 0.05) between treatments.

The mean root (0.97 kg ha^−1^), and straw (10.5 kg ha^−1^) and seed (23.1 kg ha^−1^) P concentration of maize were significantly (p ≤ 0.05) higher in SM and SI, respectively than other treatments (Fig. 3a–c). Additionally, the mean P concentration in root (1.2 kg ha^−1^), straw (11.3 kg ha^−1^) and seed (18.9 kg ha^−1^) was found considerably (p ≤ 0.05) higher in SS than those of under SI and SII in MS_R_ (Fig. 3d–f), and showed the increasing trend as the gap between maize and soybean rows (from SII to SI) increased under MS_R_. On average, relative to SI planting pattern, P concentration in root, straw and seed was increased by 68, 54, and 67% at Yaan, Renshou and Lezhi, respectively in SII in both study years. Furthermore, locations also showed the significant effects on root, straw, and seed P concentrations of maize and soybean. The mean highest P concentration in root (1.2 kg ha^−1^) and seed (24.3 kg ha^−1^), and straw (10.8 kg ha^−1^) of maize was obtained at Renshou and Yaan, respectively, and the average maximum root (0.9 kg ha^−1^), straw (8.7 kg ha^−1^), and seed (16.7 kg ha^−1^) P concentration of soybean was measured at Renshou, Yaan, and Lezhi, respectively. In addition, the different planting patterns significantly (p ≤ 0.05) affected the total P accumulation in maize and soybean at all locations. The average highest total phosphorus accumulation (TPA) of maize and soybean respectively was 34.5 kg ha^−1^ in SI and 31.5 kg ha^−1^ in SS, 35.2 kg ha^−1^ at Renshou and 25.2 kg ha^−1^ at Lezhi in both study years (Table 2).

### Root, straw, and seed potassium (K) concentration of maize and soybean

**Figure 4:**
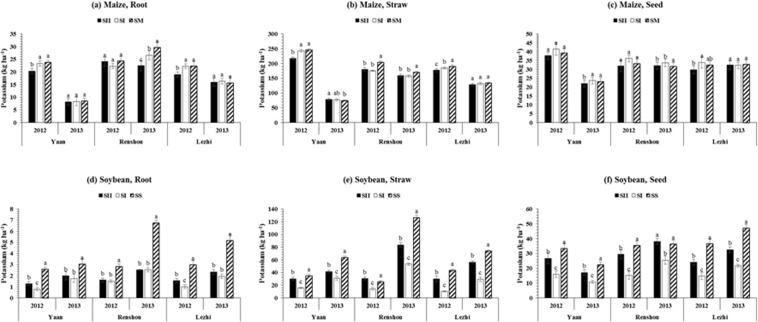
Potassium allocation in root, straw, and seed of maize and soybean at various locations (Yaan, Renshou, and Lezhi) under different planting patterns. The SI (50 cm: 50 cm) and SII (40 cm: 160 cm) represent the different planting patterns under relay-intercropping system. The SM and SS refer to sole cropping system of maize and soybean, respectively. Means are averaged over three replicates. Bars show ± standard errors, (n = 2). Within a bar, different lowercase and same letters show a significant and non-significant difference (p ≤ 0.05) between treatments.

The pattern of K distribution in different plant parts of maize and soybean is presented in (Fig. 4) for all planting patterns and locations. Large fluctuations were noted for K concentration in root, straw and seed of maize and soybean under all treatments. Maize showed mean maximum K concentrations in their root (20.7 kg ha^−1^) and straw (169.9 kg ha^−1^), and seed (33.5 kg ha^−1^) in SM and SI, respectively (Fig. 4a–c). Whereas, the mean maximum root (3.9 kg ha^−1^), straw (61.1 kg ha^−1^) and seed (35.2 kg ha^−1^) K concentrations of soybean were recorded under SS (Fig. 4d–f) than other treatments. However, among locations, the average highest K concentration in root (24.9 kg ha^−1^ and 2.9 kg ha^−1^), straw (174.4 kg ha^−1^ and 55.5 kg ha^−1^), and seed (33.1 kg ha^−1^ and 30.1 kg ha^−1^) of maize and soybean, respectively was measured at Renshou. Overall, soybean plants under SII accumulated 56, 65 and 85% higher K in root, straw, and seed, respectively as compared to SI. Furthermore, all the planting patterns and locations significantly (p ≤ 0.05) affected the total K accumulation in maize and soybean, and the mean highest total potassium accumulation (TKA) was 222.6 and 100.2 kg ha^−1^ under SM and SS, 232.5 and 88.5 kg ha^−1^ at Renshou, in both study years (Table 2).

### Total nutrient uptake of maize and soybean

**Figure 5:**
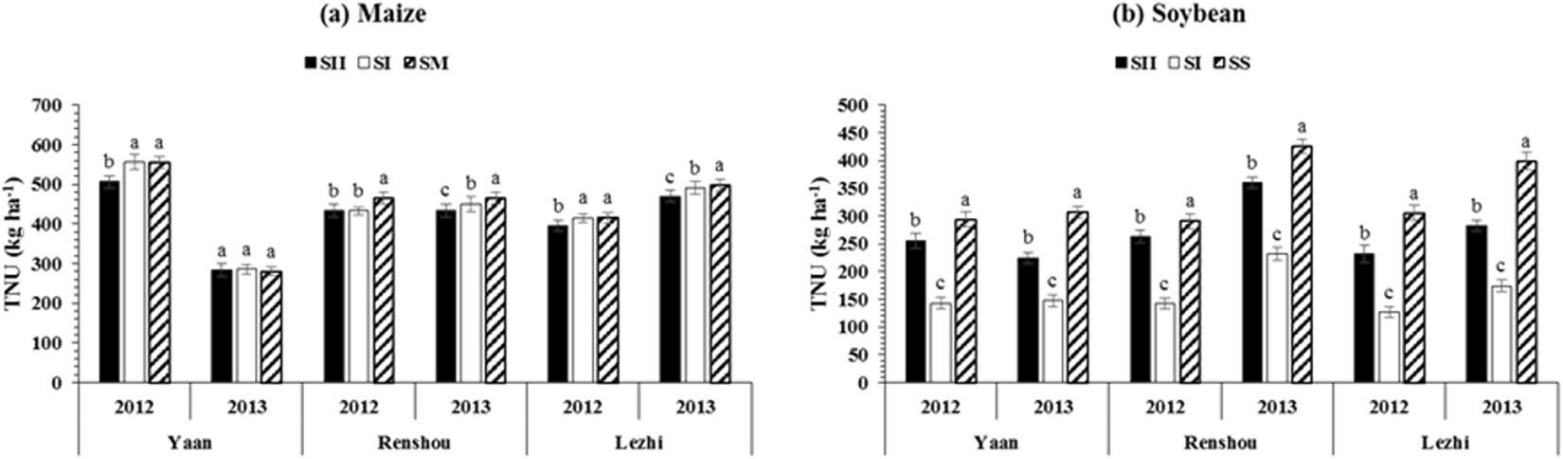
Total nutrient uptake (TPU) of maize and soybean at various locations (Yaan, Renshou, and Lezhi) under different planting patterns. The SI (50 cm: 50 cm) and SII (40 cm: 160 cm) represent the different planting patterns under relay-intercropping system. The SM and SS refer to sole cropping system of maize and soybean, respectively. Means are averaged over three replicates. Bars show ± standard errors, (n = 2). Within a bar, different lowercase and same letters show a significant and non-significant difference (p ≤ 0.05) between treatments.

The average values for total nutrient uptake (TNU) exhibited that different planting patterns and locations had significant impact on TNU of maize and soybean (Fig. 5). In this field experiment, plants under SM and SS always accumulated higher amount of nutrients than those in SI and SII under relay intercropping system. However, the planting pattern, SI (438.5 kg ha^−1^) and SII (269.7 kg ha^−1^), and location Lezhi (447.2 kg ha^−1^) and Renshou (285.2 kg ha^−1^) accumulated the highest nutrients by maize (Fig. 5a) and soybean (Fig. 5b), respectively. Furthermore, the TNU of maize showed the trend planting pattern, SM > SI > SII > and locations, Lezhi > Renshou > Yaan, and in soybean it showed planting pattern, SS > SII > SI > and locations, Renshou > Lezhi > Yaan, indicating that the wide distance between maize and soybean rows under MS_R_ considerably increased the soybean TNU (by 83% in 2012 and 57% in 2013).

### Yield, land equivalent ratio, and competition ratio of maize and soybean

Different planting patterns and locations led to significant (p ≤ 0.05) differences in seed yields, harvest index and partial land equivalent ratio (LER) of maize and soybean (Table 3). By averaging the two years data, we observed that the seed yield of maize in relay intercropping planting patterns was 95% in SII - 99% in SI of that under SM; but the planting pattern showed non-significant differences for harvest index (HI) of maize. Among locations, maximum seed yield (8021.5 kg ha^−1^) and harvest index (49.6%) of maize were found at Lezhi and Yaan, respectively. In addition, with the increased distance between maize rows (SI), the partial LER value of maize also increased, and the maximum LERm (1.0) was noticed at Renshou. Soybean seed yield depicted the significant (p ≤ 0.05) response to all treatments and locations. The mean maximum seed yield of soybean was noted in SS (2012.0 kg ha^−1^) followed by SII (1721.5 kg ha^−1^) and SI (992.4 kg ha^−1^). However, among locations, the average highest seed yields of soybean were recorded for Renshou (1640.0 kg ha^−1^) and lowest for Yaan (1492.9 kg ha^−1^) in both years. Planting pattern also showed the significant (p ≤ 0.05) effect on HI of soybean, and the mean maximum (35%) HI was measured in SII followed by SI (33%) and SS (30%). The average value of HI for locations revealed that Yaan (34%) had produced the highest value followed by Lezhi (33%) and Renshou (32%). Furthermore, with the increased distance for planting soybean between maize and soybean rows (SII) under MS_R_, the maximum partial LER (0.88) and CR (0.92) values of soybean were found in SII, among locations the highest LERs (0.80) and CRs (0.81) values were noticed at Renshou and Yaan, respectively. Overall, the LER under different planting patterns and locations ranged from 1.51 to 1.84, and SII produced the highest LER (1.81) at Renshou in both study years.

**Table 3:** Effect of different planting patterns on competition ratio (CR) and land equivalent ratio (LER) of maize and soybean at different locations under relay intercropping system.

Years	Treatments		Maize	Soybean		LER
			LERm	CRs	LERs	
2012	Planting Patterns	SI	1.00	0.46b	0.45	1.45b
		SII	0.96	0.82a	0.80	1.79a
	LSD (0.05)		NS	0.06	0.08	0.06
	Locations	Yaan	1.00	0.68	0.68	1.66
		Renshou	0.97	0.63	0.60	1.60
		Lezhi	0.98	0.61	0.60	1.60
	LSD (0.05)		NS	NS	NS	NS
2013	Planting Patterns	SI	1.00	0.56b	0.55b	1.55b
		SII	0.93	1.02a	0.98a	1.90a
	LSD (0.05)		0.03	0.03	0.04	0.02
	Locations	Yaan	0.92b	0.93a	0.87a	1.77b
		Renshou	1.05a	0.94a	0.95a	2.00a
		Lezhi	0.93b	0.50b	0.48b	1.42c
	LSD (0.05)		0.06	0.14	0.15	0.07

The SI (50 cm: 50 cm) and SII (40 cm: 160 cm) represent the different planting patterns under relay-intercropping system. The SM and SS refer to sole cropping system of maize and soybean, respectively. The LERm and LERs represent the land equivalent ratio of maize and soybean, respectively, and CRs refer to competition ratio of soybean. Means are averaged over three replicates. Means do not share the same letters in the column differ significantly at p ≤ 0.05; NS = Non-significant.

## Discussion

Higher nutrient acquisition under intercropping system confirmed the advantage of this system over sole cropping which resulted to highest dry matter accumulation (DMA) and yield. Since different planting patterns significantly influence the light environment under intercropping systems, and mutual shading considerably affects the productivity of intercrops because sun-light plays a vital role in increasing the photosynthetic rate and biomass yield^29,30^. In our experiment, different planting patterns exhibited a significant effect on the DMA of maize and soybean, and highest DMA of maize and soybean was recorded in SI and SII, respectively that was probably due to the optimum planting space, light availability and nutrients availability for maize and soybean. Our findings are similar to the previously published literature in which they concluded that DMA in maize and soybean under MS_R_ significantly affected by changing the planting pattern and light interception^9,11^. Importantly, the SII planting pattern significantly increased the soybean DMA by 61, 57, and 57% and decreased the maize DMA by 3, 3, and 5% at Yaan, Renshou, and Lezhi, respectively, maize showed small decrease in DMA as compared to a gain of dry matter by soybean plants (Table 1), results were consistent with previous researches^10^. Furthermore, the maximum DMA of maize and soybean in both study years would have been due to the adequate uptake of major nutrients^31^ during the life period of crops because improved major nutrient uptake directly increased the intercrop dry matter production^3^. Among locations, the highest DMA at Renshou as compared to Yaan and Lezhi was may be due to the favorable cropping conditions i.e. enough rainfall and temperature during the life cycle of maize and soybean (Table 2), could easily elucidate the differences in DMA of intercrop species at various locations^32^.

Cereal and legume planting together is a common agricultural phenomenon in many parts of the world^33^. However, less consideration has been given to major nutrient behavior among plant parts of intercrop species in their intercropping mixtures. In this study, nitrogen, phosphorus, and potassium concentrations among plant parts of maize and soybean varied with the planting patterns and locations, but their accumulation patterns were different in all treatments. Different planting patterns significantly affected the root, straw, and seed nitrogen concentration of maize and soybean, which might be linked to the high and low nitrogen requirement of maize and soybean, respectively in all three plant parts in different planting conditions^34,35^. In cereal-legume intercropping systems, it is possible that the maize (cereal) accumulated nitrogen that was released by soybean (legume) root and root nodule-system, resulting from efficient utilization of nutrients^36–39^. Additionally, in our experiment nitrogen concentration in roots, straw, and seeds of maize and soybean in sole maize (SM) and sole soybean (SS) was higher as compared to those plants under SI and SII at all locations, suggesting that intercropping mixtures reduced the nitrogen accumulation amount in root, straw, and seed (Fig. 2). Similar to our results, Gou *et al*., (2018) reported the decreased nitrogen and phosphorus concentration in maize under relay intercropping system than sole cropping system^27^. However, the nitrogen concentration differences between relay-cropped maize and sole cropped maize were smaller as compared to soybean (Fig. 2a–c). It might be caused by the strong nitrogen acquisition ability of neighboring deep-root cereal species maize, whose root hair system intermingled with the soybean roots and acquired more legume-derived nitrogen^40,41^. Importantly, soybean plants significantly accumulated higher amounts of nitrogen in SII than SI which is might be due to the better growing conditions and availability of nutrients because increasing distance between maize and soybean rows reduced the maize shade^23^, increased the light transmittance on soybean canopy^11^ and decreased competition for the accumulation of nitrogen^8^ which significantly enhanced the concentration of N in soybean under MS_R_.

Phosphorus concentrations among different plant parts of soybean plants under MS_R_ had been reduced as compared with its sole cropping plants (Fig. 3d–f), however, these differences were decreased under SII than SI planting pattern. Likely, there were two main processes that might had influenced the phosphorus concentration in soybean plants as its concentration decreased under the intercropping systems: (1) application of phosphorus-containing fertilizers tended to enhance phosphorus availability, and (2) limited phosphorus availability from soil and fertilizer was equally utilized by every single plant of soybean. Consequently, increased number of plants under intercropping conditions would reduce the phosphorus reallocation, limiting soybean phosphorus acquisition and concentration in different plant parts of soybean^42^. Similar findings were found for citrus-soybean intercropping system, where phosphorus accumulation and distribution among the different plant parts of soybean were reduced as compared to their corresponding monoculture^43^. These dissimilarities with what was estimated that phosphorus uptake would be increased in both cereals and legumes in their intercropping stands because pH decreased in soil-rhizosphere, and phosphorus activities in roots (i.e. phosphatase activities in soil-rhizosphere) and soil and soil-rhizosphere phosphorus concentration increased^1,44^. Unfortunately, in this experiment, we have not determined the phosphatase activity and soil-rhizosphere pH in the roots and soil. Interestingly, the differences of total phosphorus uptake (Table 3) and distribution among different plant parts of maize under SI and SII in MS_R_ and SM decreased (Fig. 3a–c) as the distance between maize and soybean rows decreased (SI), indicating the reduced interspecific competition which significantly increased the phosphorus uptake^45^. Similarly, it may also be caused due to the border row effects in MS_R_ because previously scientists have confirmed that the border row wheat plants had higher nitrogen and phosphorus uptake than those of under sole cropping system^3,25^. On average, soybean plants in SII accumulated 63% of higher phosphorus (TPU) as compared to SI where soybean plants experienced severe shading conditions which showed the direct relationship between light intensity and phosphorus uptake because several scientists have proved that shading conditions significantly reduced the phosphorus uptake ability in crops^46,47^. Therefore, higher accumulation of phosphorus in maize and soybean is might be due the improved light conditions and growing space for intercrop species under SII (narrow wide row planting pattern) than SI.

In this paper, we also measured the differences of potassium uptake and distribution in different plant parts of maize and soybean under MS_R_ in response to different planting patterns at different locations (Fig. 4). The potassium concentration in maize plants was generally higher as compared to soybean plants. However, the smaller amount of potassium uptake was recorded in relay-cropped maize and soybean plants than those with under SM and SS, which indicates the interspecific competition between maize and soybean. On contrary to our findings, higher potassium uptake in intercropped wheat was reported under wheat-maize intercropping system^3^. The different planting patterns significantly affected the potassium uptake in soybean, and planting pattern SII increased the uptake of potassium in soybean. It might be due to the better planting space and arrangement that enhanced the potassium accumulation ability of soybean plants by ameliorating the negative effects of competition and maize shading^4^. Results of our experiment confirm that different planting patterns significantly affected the nutrient uptake ability of intercrop species, differences in nutrient acquisition (K) between MS_R_ and sole cropping system by soybean plants (Fig. 4d–f) was larger as compared to maize plants (Fig. 4a–c). Overall, the higher N and P concentrations in seed than in other parts and the higher K concentration in straw than root and seed of maize and soybean, could be described by the competition among plant parts for N, P, and K demand for plant growth, and the advantage of that plant part may have for being adjacent to source of nutrient. This is because N, P, and K are distributed to distant parts after the request of adjacent parts to a nutrient source are met^45^. Conversely, this challenges the results from shrubs, where the nutrient content in roots enhanced in equivalent with the stem and leaves^45^. Nutrient distribution is also controlled by the crop and plant part request for nitrogen-rich proteins and phosphorus-rich RNAs^48^. Extra nitrogen and phosphorus distribution to the leaf are important to maintain the photosynthesis process efficiency and photo-assimilate remobilization^49^. In arid areas (or drought years), a high nitrogen content in leaf may provide adaptation to water scarce conditions and competition by exploiting the greater sunlight availability^45,49,50^.

**Figure 6:**
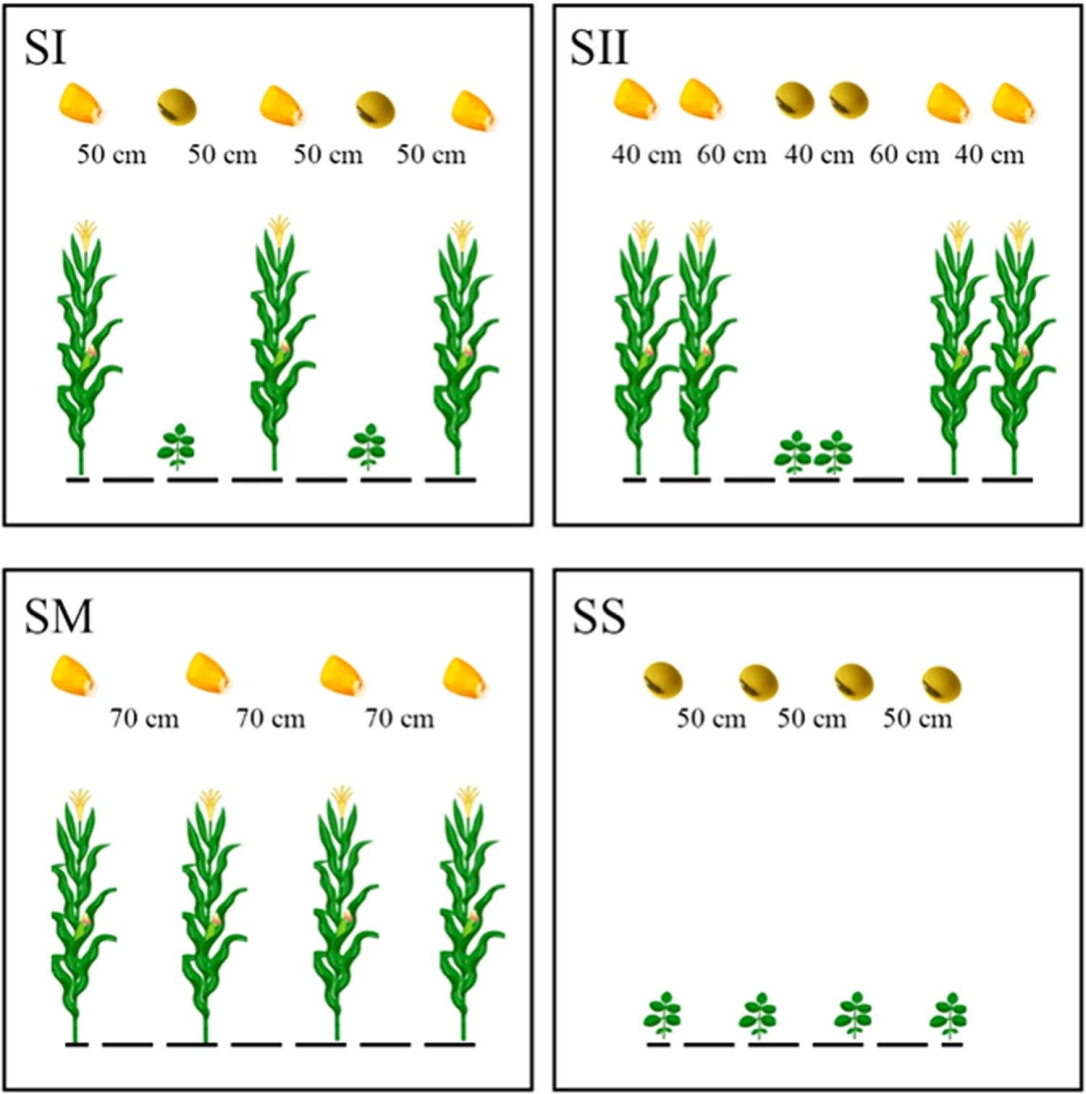
Field layout of different planting patterns of relay-intercropped maize soybean: SI (traditional single row relay-intercropping system of maize and soybean, one row of soybean relay-intercropped with one row of maize, the row to row distance between maize and soybean rows was 50 cm), SII (modern double row relay-intercropping system of maize and soybean, two rows of soybean relay-intercropped with two rows of maize, maize row to row distance was 40 cm, soybean row to row distance was 40 cm, distance between the maize and soybean rows was 60 cm), SM (sole maize, row to row distance was 70 cm), and SS (sole soybean, row to row distance was 50 cm).

Previously, it has been reported that nutrient uptake and nutrient use efficiency were greatly reduced by decrease in light intensity^47,51–53^. Therefore, these experiments were carried out to determine the effects of different planting patterns on total nutrient uptake of maize and soybean at different locations. The findings of the present study reported here demonstrate that total nutrient uptake (TNU) in soybean was increased by increasing the distance between maize and soybean rows under MS_R_. Specifically, soybean plants grown under SII accumulated 18% higher TNU than those plants in SI while SII decreased the TNU of maize by 4% than SI in both years (Fig. 6). This enhanced TNU in soybean under narrow-wide row planting pattern (SII) increased both the dry matter production (Table 1) and distribution in plant organs (Fig. 1). Based on these results, non-exclusive, hypotheses might be postulated for the uptake of nutrients in MS_R_ that improvement in light environment (data not shown) in SII enhances the photosynthesis, carbon fixation, and plant growth rate of soybean plants. To maintain these processes at optimum level soybean plants uptake higher amount of N, P, and K in SII than SI. Results were similar with previous findings in which scientists reported that nutrients uptake increased in lupin (*Lupinus albus* L.) with increasing the light intensity^54^. Moreover, higher light (photosynthetically active radiations) intensity improved the distribution pattern of major nutrients in crops which significantly increased the dry matter production^47^.

The effect of different planting patterns on yield of maize and soybean under MS_R_, SM, and SS at different locations was evaluated, and the results of our study revealed that the total yield of maize and soybean were significantly (p ≤ 0.05) increased with optimum strip width arrangement SII at all locations (Table 3). This increase might be due to the optimum availability and utilization of major nutrients (N, P, and K) as these nutrients increased the yield and yield related parameters^8,55,56^. Further, our results exhibited that increasing the distance between maize and soybean rows i.e. 40:160 (SII) improved the seed yield of soybean at all locations due to the substantial increase in DMA and nutrient uptake by maintaining the maize seed yield. In the past study, researchers have reported that balanced fertilization and optimum uptake of major nutrient significantly increased the intercrop yields under MS_R_^24^. Total LER values were always higher than one in both planting patterns in MS_R_ at all locations, which exhibits the yield benefit of MS_R_ over sole cropping due to the better utilization of land and environmental resources for crop growth and development^12^. Particularly, the mean values of LER under SI and SII were 1.84 and 1.51 (Table 3), respectively, which means that 50 to 80% of extra farmland will be needed by the sole cropping of maize and soybean to equal the seed yields of relay-intercropping system, showing intercrops land use efficiency as compared with sole crops^10,57,58^. These results are in line with those of Yang *et al*., (2017) who reported the MS_R_ advantage with narrow wide row planting pattern^11^. Similarly, Liu *et al*., (2017) reported higher LER values 1.3–1.4 in narrow wide row intercropping system of maize and soybean^59^. In addition, less distance (52 cm) between maize and soybean rows negatively affected the light interception at the soybean canopy^9,60,61^, therefore it is an effective method to ameliorate the negative effects of maize shade by increasing the distance (SII, 40:160) between maize and soybean rows for planting soybean, which resulted in higher light interception^9^, dry matter^14^ and seed yield production of soybean and ultimately the total LER of relay-intercropping system. In addition, the partial values of CR clearly showed maize as the dominant crop species under MS_R_ (Table 3). Similarly, in many previous investigations, it has been proved that the values of CR were always higher than soybean^12,59^. Moreover, higher competitive ability of maize crop to exploit and use available resources i.e. light, land, and water in association with soybean or groundnut or chickpea has been confirmed by other scientists^8,62,63^. Whereas, in pea-rye intercropping the partial values of CRp of legume (pea) were greater than cereal (rye)^64^, which was the different trend which we observed as cereal (maize) was more aggressive and competitive than legume (soybean).

## Conclusion

In our experiment, relay-cropped maize and soybean under different planting patterns had accumulated lower nutrient (N, P, and K) than those of under SM and SS. However, the differences in nutrient uptake and distribution among different plant parts for soybean between relay-cropped and sole cropped plants were larger than maize plants under SI and SII planting patterns. Furthermore, the high seed yield of soybean under SII in MS_R_ was a result of higher dry matter accumulation. In SII, 40:160 optimum strip width for growing maize and soybean, it is the best planting pattern to minimize the shading effect of maize on soybean at critical stages during the co-growth period i.e. vegetative stage and the start of flowering stage. Therefore, planting pattern SII can be adopted in MS_R_ to avoid sever shading of maize at flowering stage. In MS_R_ wide distance between maize and soybean rows potentially increased the land equivalent ratio (1.84) which was the result of adequate maize (182.8 kg N ha^−1^, 31.3 kg P ha^−1^, and 206.2 kg K ha^−1^) and soybean (170.5 kg N ha^−1^, 23.9 kg P ha^−1^, and 44.7 kg K ha^−1^) nutrient acquisition in SII (Table 2). In addition, our experiment suggested that for the sustainability and productivity of maize soybean relay-intercropping system, narrow wide row strip width planting pattern (SII) has to be used to improve the competition indices of maize and soybean under MS_R_. Moreover, by the selection of optimum planting pattern (SII; 40:160), we can achieve higher seed yield and economic return under MS_R_. Furthermore, planning the long-term field observations to understand the impact of various planting pattern and intercrop combinations on the nutrients uptake of intercrop species is an important direction for future research.

## Methods

### Experimental sites

Three experimental sites, Yaan, Renshou, and Lezhi, with different altitude levels and rainfall characteristics, were selected for this experiment. The field experiment did not include any protected or endangered species and no permissions were needed for the selected locations. All the experiments were conducted by following the institutional rules and regulations of Sichuan Agricultural University, China. Yaan (29°59′N, 103°00′E, altitude 620 m elevation) has a humid climatic condition with mean annual temperature of 16.2 °C and rainfall 1200 mm. The subtropical and humid site Renshou (30°16′4”N, 104°12′53”E, altitude 482 m elevation), has average rainfall of 1009.4 mm and temperature 17.4 °C. Lezhi (30°30′4′′N and 104°45′2′′E, altitude 446 m elevation) has monsoon humid, with mean rainfall of 949.4 mm and temperature of 16.7 °C. Weather data which includes monthly rainfall, average temperature, humidity and wind speed during the planting seasons from 2012 to 2013 are shown in Table 4. The physiochemical characteristics of soil at Yaan pH = 6.6, organic matter = 29.8 g kg^−1^, total N = 1.6 g kg^−1^, total P = 1.28 g kg^−1^, total K = 16.3 g kg^−1^, available N = 317.1 mg kg^−1^, available P = 42.2 mg kg^−1^, and available K = 382.1 mg kg^−1^, at Renshou pH = 6.8, organic matter = 17.3 g kg^−1^, total N = 0.9 g kg^−1^, total P = 0.5 g kg^−1^, total K = 14.3 g kg^−1^, available N = 77.4 mg kg^−1^, available P = 10.2 mg kg^−1^, and available K = 197.1 mg kg^−1^, and at Lezhi were pH = 6.8, organic matter = 11.1 g kg^−1^, total N = 1.0 g kg^−1^, total P = 1.4 g kg^−1^, total K = 17.9 g kg^−1^, available N = 165.1 mg kg^−1^, available P = 4.9 mg kg^−1^, and available K = 391.1 mg kg^−1^, in the 0–20 cm soil layer.

**Table 4:** Monthly rainfall, average temperature, humidity, and wind speed from March to October in the growing seasons of 2012 and 2013.

Years	Month	Locations					
		Yaan		Renshou		Lezhi	
		Rainfall (mm)	Average T (°C)	Rainfall (mm)	Average T (°C)	Rainfall (mm)	Average T (°C)
2012	March	15.3	14.2	6.2	16.2	21.9	16.1
	April	97.8	20.1	81.5	21.5	43.2	23.4
	May	153.4	24.2	117.9	23.3	75.3	24.7
	June	185.1	25.8	134.4	26.7	132.5	24.5
	July	364.6	25.2	285.3	25.1	159.1	26.2
	August	225.9	26.4	279.1	24.5	167.4	27.3
	September	217.3	20.3	153.7	21.3	106.3	22.7
	October	58.5	17.3	94.6	20.7	45.8	18.4
March–October		1317.9	21.7	1152.7	22.4	751.5	22.9
2013	March	57.1	11.7	64.5	17.4	30.4	17.3
	April	80.5	17.6	71.9	17.1	60.7	21.5
	May	236.7	21.2	189.3	23.5	101.5	22.7
	June	260.8	24.1	256.1	25.7	158.3	26.5
	July	367.9	24.5	335.5	26.3	179.1	27.9
	August	415.6	25.4	294.3	25.9	196.9	23.2
	September	163.5	21.2	83.1	22.4	135.2	22.3
	October	116.4	18.6	35.6	19.5	53.7	19.1
March–October		1698.5	20.5	1330.3	22.2	915.8	22.5

### Planting material and experimental details

The maize genotype ‘Chuandan-418 (semi-compact)’ and the soybean genotype ‘Nandou-12 (shade resistant)’ were selected for the experiments. This field study consisted of four different planting pattern arrangements (Fig. 6), described as follows, sole maize (SM, row to row distance was 70 cm), sole soybean (SS, row to row distance was 50 cm), single row relay-intercropping system of maize and soybean (SI, one row of soybean relay-intercropped with one row of maize, the row to row distance between maize and soybean rows was 50 cm), and double row relay-intercropping system of maize and soybean (SII, two rows of soybean relay-intercropped with two rows of maize, maize row to row distance was 40 cm, soybean row to row distance was 40 cm, distance between the maize and soybean rows was 60 cm). Every experimental block was 6 m long with three strips. The field experiments were laid out using a randomized complete block design with three replicates. Both varieties were over seeded and thinned to maintain a uniform planting density of 6 and 10 plants m^−2^ for maize and soybean, respectively in relay-intercropping system, and similar planting density was kept in sole cropping system of maize and soybean. The maize crop was sown in the second week of April in 2012 and 2013, and harvested in the first week of August 2012 and 2013. Soybean was sown on in the second week of June 2012 and 2013 and harvested in the last week of October 2012 and 2013. All plots were treated with basal fertilizer. Basal nitrogen (N) at 135 kg ha^−1^ as urea, phosphorus (P) at 40 kg ha^−1^ as calcium superphosphate, and potassium (K) at 10 kg ha^−1^ as potassium sulfate were applied at the time of sowing in intercropped and sole-cropped maize. At the V_6_ stage of maize, the second dose of N was applied at 75 kg ha^−1^ as urea in all plots. The N, P, and K at 75, 40, and 4 kg ha^−1^ as urea, calcium superphosphate, and potassium sulfate, respectively were basally applied for soybean at the time of soybean sowing. Other farming measures were used according to the farmer’s practices.

### Sampling and measurements

#### Dry matter accumulation and partioning

Ten consecutive maize and soybean plants, excluding the five border plants, were destructively sampled from all experimental blocks at maturity stage during both years and locations for the determination of total dry matter accumulation (DMA) and partitioning in different plant parts of maize and soybean. Then all the sampled plants were divided into different plant parts of maize (root, straw (leaves + stem), and seed) and soybean (root, straw (leaves + stem + pod cover), and seed) placed in oven for one hour at 80 °C to kill the fresh-tissues and then dried at 65 °C to obtain constant weight before weighing of each plant part of maize and soybean for total dry matter accumulation and partitioning analysis.

#### Nutrient uptake in maize and soybean

At maturity of maize and soybean, plant samples were collected from the central rows of each experimental block and location, divided into root, straw, and seed of maize and soybean. Then all the maize and soybean samples were dried in the oven at 80 °C for 96 hours to attain constant dry weights. Then dry matter of each plant part was ground using a ring-mill through hundred-mesh and the nitrogen (N) content of maize and soybean plant samples was measured by using the Kjeldahl method^3^, the phosphorus (P) content of maize and soybean plant samples was determined by using the vanadomolybdate procedure^55^, and the potassium (K) content of maize and soybean plant samples were estimated by following the previously described method^65^. The N, P, and K content in each organ of maize and soybean plants were measured by multiplying the total dry matter of each plant organ with the N, P, and K content and calculated in a kg ha^−13^. The total N accumulation (TNA), total P accumulation (TPA), and total K accumulation (TKA) were calculated from the summation of the nitrogen, phosphorus, and potassium in all plant parts^56^. Additionally, total nutrient uptake (TNU) was measured from the summation of the TNA, TPA, and TKA in maize and soybean.

#### Yield and competition parameters

In this experiment, at the time of maturity, four m^−2^ plants of maize and soybean were manually harvested from the central rows of each experimental block using sickle at ground level. Then these sampled plants were dried for ten days. The dried maize and soybean plants were threshed manually and weighed to determine the intercropped and sole cropped maize and soybean seed yields of every plant and then converted into kg ha^−1^. In addition, harvest index (HI) was estimated by dividing the seed weight per plant by the dry matter weight of each plant at physiological maturity. The relay-intercropping advantage and competition effects between two different crop species grown in mixture were determined by using several competition parameters as follows: the LER was applied as the standard for intercropping advantage because both maize and soybean were the preferred crop species in relay-intercropping system. One is considered as the crucial LER value because if the value of LER is greater than one then it means that intercropping system favors the crop growth and yield of intercropped species, while if LER value is less than one then that intercropping system negatively reduces the growth and yield of intercropped species^66^. LER was determined as:

1 $$LER=LERm/LERs$$

2 $$LER=Ymr/Yms$$

3 $$LER=Ymr/Yms$$

where Yms and Yss are the seed yields of maize and soybean in sole cropping system, respectively, and Ymr and Ysr are the seed yields of maize and soybean, respectively under relay-intercropping system. The competition ratio (CR) is another parameter to investigate the competition between two crop species. The CRs is determined by using the following formulas:

4 $$CRm=(LERm/LERs)\times (Zsr/Zmr)$$

5 $$CRs=1/CRm$$

where Zsr and Zmr are the soybean and maize sown proportion area in relay-intercropping system, respectively. When the value of CRs is higher than ‘one’ suggested the competitive ability of soybean greater than maize.

### Statistical analysis

All the measured data for all parameter was analyzed using Statistix 8.1. An ANOVA (analysis of variance) technique was used to confirm the overall significance of data. The least significance difference (LSD) test was applied to compare the means at 5 percent probability level^67^. In addition, Microsoft Excel program was used for the graphical presentation of data using standard error (±SE).

## Data Availability

The datasets generated during and/or analyzed during the current study are available from the corresponding author on reasonable request.
